# Supplementary material for: Understanding painful versus non-painful dental pain in female and male patients: A transcriptomic analysis of human biopsies
Source: PLoS One. 2023 Sep 21;18(9):e0291724. doi: 10.1371/journal.pone.0291724 (PMC10513205; doi:10.1371/journal.pone.0291724)
Supplement: S1 Table — (DOCX) [file pone.0291724.s001.docx]

**S1 Table**

| **Genes Upregulated in Symptomatic Males Compared to Asymptomatic Males** | |
| --- | --- |
| **Genes** | **Function** |
| VNN1 | Immune Response |
| IL10 | Immune Response |
| GNLY | Immune Response |
| PLK2 | Multiple Functions |
| GUCY1A3 | Other |
| TDO2 | Other |

S1 Table
