# Supplementary material for: Understanding painful versus non-painful dental pain in female and male patients: A transcriptomic analysis of human biopsies
Source: PLoS One. 2023 Sep 21;18(9):e0291724. doi: 10.1371/journal.pone.0291724 (PMC10513205; doi:10.1371/journal.pone.0291724)
Supplement: S2 Table — (DOCX) [file pone.0291724.s002.docx]

**S2 Table**

| **Genes Downregulated in Symptomatic Males Compared to Asymptomatic Males** | |
| --- | --- |
| **Genes** | **Function** |
| TWIST2 | Immune Response |
| SAA2 | Immune Response |
| TIMP3 | Immune Response |
| CXCL14 | Immune Response |
| CCL22 | Immune Response |
| SOD3 | Repair |
| IGF2 | Repair/Regeneration |
| ITGBL1 | Cell Adhesion |
| FBLN5 | Vascular and Cell Adhesion |
| COL8A1 | Extracellular Matrix and Vascular |
| CORIN | Neural |
| LEPR | Neural and Bone Metabolism |
| MOXD1 | Other |
| OMD | Other |
| DIRAS3 | Other |
| CPXM2 | Other |
| LTBP2 | Other |
| DKK3 | Other |

S2 Table
