# Supplementary material for: Understanding painful versus non-painful dental pain in female and male patients: A transcriptomic analysis of human biopsies
Source: PLoS One. 2023 Sep 21;18(9):e0291724. doi: 10.1371/journal.pone.0291724 (PMC10513205; doi:10.1371/journal.pone.0291724)
Supplement: S3 Table — (DOCX) [file pone.0291724.s003.docx]

**S3 Table**

| **Genes Upregulated in Symptomatic Females Compared to Asymptomatic Females** | |
| --- | --- |
| **Genes** | **Function** |
| PVRIG | Immune response |
| CCR7 | Immune Response |
| ERAP2 | Immune Response |
| WFDC2 | Multiple Functions |
| DCLK1 | Neural |
| F3 | Vascular |
| LAMA2 | Extracellular Matrix |
| HERC2P2 | Other |
| LOC100130231 | Other |
| LOC144571 | Other |
| SPOCK2 | Other |

S3 Table
