# Supplementary material for: Understanding painful versus non-painful dental pain in female and male patients: A transcriptomic analysis of human biopsies
Source: PLoS One. 2023 Sep 21;18(9):e0291724. doi: 10.1371/journal.pone.0291724 (PMC10513205; doi:10.1371/journal.pone.0291724)
Supplement: S4 Table — (DOCX) [file pone.0291724.s004.docx]

**S4 Table**

| **Genes Downregulated in Symptomatic Females Compared to Asymptomatic Females** | |
| --- | --- |
| **Genes** | **Function** |
| STAP1 | Immune Response |
| DAPK2 | Immune Response |
| MMP13 | Extracellular Matrix |
| SNORA53 | Other |
| SNORD33 | Other |
| SQLE | Other |
| HIST1H3B | Other |
| IBSP | Other |
| SNORD110 | Other |
| RNU12 | Other |
| HIST1H3C | Other |
| HIST1H2BG | Other |
| ADCYAP1 | Other |
| C3orf70 | Other |

S4 Table
