# Supplementary material for: Understanding painful versus non-painful dental pain in female and male patients: A transcriptomic analysis of human biopsies
Source: PLoS One. 2023 Sep 21;18(9):e0291724. doi: 10.1371/journal.pone.0291724 (PMC10513205; doi:10.1371/journal.pone.0291724)
Supplement: S5 Table — (DOCX) [file pone.0291724.s005.docx]

**S5 Table**

| **Genes Upregulated in Symptomatic Females Compared to Symptomatic Males** | |
| --- | --- |
| **Genes** | **Function** |
| ERAP2 | Immune Response |
| CHIT1 | Immune Response |
| C3 | Immune Response |
| SIGLEC1 | Immune Response |
| SLC18A2 | Neural |
| CADM3 | Cell Adhesion |
| CTGF | Cell Adhesion |
| LRRC15 | Cell adhesion |
| ABI3BP | Extracellular Matrix |
| CHRDL2 | Bone Metabolism |
| SOD3 | Repair |
| SIK1 | Other |
| CPZ | Other |
| FOLR2 | Other |
| ACAB | Other |

S5 Table
