# Supplementary material for: Understanding painful versus non-painful dental pain in female and male patients: A transcriptomic analysis of human biopsies
Source: PLoS One. 2023 Sep 21;18(9):e0291724. doi: 10.1371/journal.pone.0291724 (PMC10513205; doi:10.1371/journal.pone.0291724)
Supplement: S6 Table — (DOCX) [file pone.0291724.s006.docx]

**S6 Table**

| **Genes Downregulated in Symptomatic Females Compared to Symptomatic Males** | |
| --- | --- |
| **Genes** | **Function** |
| PDPN | Immune Response |
| VNN1 | Immune Response |
| IL1B | Immune Response |
| MIR650 | Multiple Functions |
| LRRK2 | Neural |
| HIST1H3J | Other |
| HIST1H2AB | Other |
| PGM2 | Other |
| PGD | Other |
| CYorf15A | Other |
| CYorf15B | Other |

S6 Table
