# Supplementary material for: Understanding painful versus non-painful dental pain in female and male patients: A transcriptomic analysis of human biopsies
Source: PLoS One. 2023 Sep 21;18(9):e0291724. doi: 10.1371/journal.pone.0291724 (PMC10513205; doi:10.1371/journal.pone.0291724)
Supplement: S7 Table — (DOCX) [file pone.0291724.s007.docx]

**S7 Table**

| **Genes Upregulated in Asymptomatic Females Compared to Asymptomatic Males** | |
| --- | --- |
| **Genes** | **Function** |
| HLA-J | Immune Response |
| CX3CR1 | Immune Response |
| ATP10B | Neural |
| COCH | Extracellular Matrix |
| RNU12 | Other |
| RPL9 | Other |

S7 Table
