# Supplementary material for: Understanding painful versus non-painful dental pain in female and male patients: A transcriptomic analysis of human biopsies
Source: PLoS One. 2023 Sep 21;18(9):e0291724. doi: 10.1371/journal.pone.0291724 (PMC10513205; doi:10.1371/journal.pone.0291724)
Supplement: S8 Table — (DOCX) [file pone.0291724.s008.docx]

**S8 Table**

| **Genes Downregulated in Asymptomatic Females Compared to Asymptomatic Males** | |
| --- | --- |
| **Genes** | **Function** |
| CCL22 | Immune Response |
| SERPINE1 | Repair |
| SDC4 | Extracellular Matrix |
| COL9A3 | Extracellular Matrix |
| CPE | Neural |
| CYorf15A | Other |
| SNORD18C | Other |
| CYorf15B | Other |
| FOXQ1 | Other |
| HERC2P2 | Other |

S8 Table
