# Supplementary material for: Understanding painful versus non-painful dental pain in female and male patients: A transcriptomic analysis of human biopsies
Source: PLoS One. 2023 Sep 21;18(9):e0291724. doi: 10.1371/journal.pone.0291724 (PMC10513205; doi:10.1371/journal.pone.0291724)
Supplement: Attachment — Submitted filename: Response to Reviewers.docx [file pone.0291724.s009.docx]

We are very thankful for the thoughtful comments of the reviewers. Please see our responses and revisions for each comment.

Reviewer #1: This manuscript mainly reports the RNA-seq results of the periapical tissue of patients with periapical periodontitis, compares the gene expression differences between symptomatic and asymptomatic, or male and female, and uses RT-qPCR to verify key genes. There are however some concerns that need to be addressed satisfactorily by the authors before this manuscript could be considered acceptable.

1. About the Exclusion and Inclusion Criteria:
1) 12 patients were included for RNA-seq, however, I notice that the oldest was 89 years old and the youngest was 19 years old. I wonder if age is a factor that affects the RNA-seq results? The same concern applies to the race of the patients who were included.

We appreciate and concur with the reviewer's feedback regarding age and race as variables. We therefore, have conducted validation studies using RT-PCR on a selection of target genes. Our study includes a sample size of 6-10 individuals per group, encompassing a broad range of ages and both Hispanic and White for races. These additional experiments serve to support and validate the outcomes derived from the bulk RNA-seq studies. By incorporating diverse biological samples, we aim to minimize the impact of age- and race-related variability and strengthen the reliability of our findings. We have now included this point in the “Discussion” section of the manuscript.

2) There is a lack of description of the details of each patient's condition, such as the degree and scope of periapical resorption in each patient, and whether the difference in the degree of infection will also affect the RNA-seq results?

We greatly appreciate the thoughtful comment provided. In our patient cohort study, we did not specifically evaluate the size of periapical bone resorption. It is important to note that this cohort of patients are defined by chronic non-healing periapical lesions that are excised during microsurgical procedures and therefore have *chronic* periapical pathosis. Additionally, no studies have established a direct link between the size or chronicity of periapical lesions and the pain phenotype, suggesting a lack of correlation. However, this warrants a future study. We have now included and clarified this point in the “Experimental

Procedures” section of the manuscript.

2. About the sample collection:
The author mentions “3-8 mm3 biopsies were collected” in the manuscript, but lacks the description of details, for example, were the biopsies taken from the apical foramen or from the center of the lesion? Or randomly take 3-8mm3 from the diseased tissue removed during the surgery?

Periapical lesions were collected in its entirety. We have specified this point in the “Experimental Procedures” section. However, patients that requested a pathology report had part of the lesion submitted to pathology for a biopsy report.

3. About the RT-PCR:
1) In the section of Results, the authors mention that “Using biological samples (N=6-12) entirely different from the ones used in generating RNA sequencing datasets, we conducted validation studies using RT-PCR.” In the Methods section the authors do not mention these details.

We now provide demographic details for each sample used for RT-PCR validation studies in the “Experimental Procedures” section.

2) Do the samples for PCR meet the same exclusion and inclusion criteria? I think the patient information for PCR should also be listed as a table in the methods section.

Yes, all patient samples for RT-PCR were collected using the same inclusion and exclusion criteria. We have now state this in the “Experimental Procedures” section.

4. About the Symptomatic or Asymptomatic patient:
1) The authors define whether symptoms are present through palpation and percussion, and patients with mechanical allodynia to palpation or percussion are considered symptomatic patients. Table 1 should also indicate whether patients with mechanical allodynia to both examinations or only one examination.

2) Were all examinations performed by the same person, and are the standards the same? The authors should add the description of the details of the examination.

Clinical exams were performed by multiple providers. However, each provider was calibrated prior to the start of the study with respect to clinical diagnosis and periapical lesion excision. Tissue procurement and storage was also standardized for all research personnel. We add this detail now in the “Experimental Procedures” section.

Reviewer #2: This is a well conducted study, which is likely to have a great impact once published.
there are some minor comments: Fig 1-A and 1B and Fig 4: legends are not visible, please enlarge/bold for further clarity for the reader.

We apologize for the poor quality of figures for Fig1. and 4. We have now included new figures for Fig. 1 and 4.

Please make sure all the abbreviations are defined at their first appearance in the manuscript.

Thank you and we now have proofread the manuscript for these.
