# Supplementary material for: Understanding painful versus non-painful dental pain in female and male patients: A transcriptomic analysis of human biopsies
Source: PLoS One. 2023 Sep 21;18(9):e0291724. doi: 10.1371/journal.pone.0291724 (PMC10513205; doi:10.1371/journal.pone.0291724)
Supplement: Attachment — Submitted filename: Response to Reviewers .docx [file pone.0291724.s010.docx]

We thank the reviewer for the positive feedback and questions. Please see our responses in blue font.

Reviewer #3: this is a well written paper. my main concerns are:
1. In the main analysis of transcriptional changes all of the symptomatic males were Hispanic and the asymptomatic males were Asian and White. my concern is that the results could be due to population stratification rather than true differences in expression changes.

We appreciate this concern of the reviewer. While our initial sample cohort might appear limited in terms of racial diversity within the symptomatic male (SM) group, our samples encompassed both Hispanic and White male populations. This broad representation ensures that our findings are not confined to a singular demographic, thus enhancing the applicability and relevance of our results across different populations. This deliberate inclusivity was aimed at preventing any undue bias or overrepresentation and promoting a more comprehensive understanding of the underlying biological processes. Collectively, we have diligently taken steps to enhance the robustness and validity of our outcomes.

In our revised manuscript, we have acknowledged this within the “Discussion” section of the manuscript. It states as follows.

“A small sample size of N=3 with a wide age range and race was used for bulk RNA sequencing, which potentially reduces the generalizability of our findings. Moreover, the majority of patients declined a histopathologic assessment of the biopsy samples. Given the incidence of periapical granuloma vs. periapical abscess vs. periapical cyst, the differential cellular compositions of each of these diagnoses present a biological confound in our patient samples. However, we conducted validation studies using RT-PCR of several target genes with separate biological samples with an N=6-10 per group. This greatly increases the rigor and internal validity of our findings and minimize the impact of age, race or histopathology-related variability.”

2. In Table 2 there is no information on Asymptomatic females. Were they not included in the secondary analyses? could you please clarify?

We appreciate this question. In our validation studies, we opted for the "Convenience Sampling" method to streamline the process. This approach involved focusing on genes with a robust expression level, indicated by an RPKM (Reads Per Kilobase Million) value exceeding 10, as this threshold ensured a reliable detectable range when employing the RT-PCR technique. The symptomatic male (SM), symptomatic female (SF), and asymptomatic male (AM) groups exhibited a higher prevalence of genes meeting the RPKM criterion, making them conducive for efficient validation through RT-PCR. The accessibility and availability of samples (N=6-12) from these groups were also comparably greater in comparison to the asymptomatic female (AF) group. This difference in accessibility contributed to the expeditious validation of our bulk RNA sequencing findings. However, we acknowledge the need for future studies to encompass a a broader spectrum of genes, enhancing the comprehensiveness and reliability of our results as well as a crucial precursor to subsequent functional studies.
